# Safety and feasibility of xenon as an adjuvant to sevoflurane anaesthesia in children undergoing interventional or diagnostic cardiac catheterization: study protocol for a randomised controlled trial

**DOI:** 10.1186/s13063-015-0587-3

**Published:** 2015-03-04

**Authors:** Sarah Devroe, Jurgen Lemiere, Marc Van de Velde, Marc Gewillig, Derize Boshoff, Steffen Rex

**Affiliations:** Department of Anaesthesiology, University Hospitals of the KU Leuven, Herestraat 49, 3000 Leuven, Belgium; Department of Child and Adolescent Psychiatry, University Hospitals of the KU Leuven, Herestraat 49, 3000 Leuven, Belgium; Department of Paediatric Haemato-Oncology, University Hospitals of the KU Leuven, Herestraat 49, 3000 Leuven, Belgium; Department of Cardiovascular Sciences, KU Leuven, Herestraat 49, 3000 Leuven, Belgium; Department of Paediatric and Congenital Cardiology, University Hospitals of the KU Leuven, Herestraat 49, 3000 Leuven, Belgium

**Keywords:** Anaesthetics, Inhalation, Xenon, Sevoflurane, Congenital heart defects, Cardiac catheterization

## Abstract

**Background:**

Xenon has minimal haemodynamic side effects when compared to volatile or intravenous anaesthetics. Moreover, in *in vitro* and in animal experiments, xenon has been demonstrated to convey cardio- and neuroprotective effects. Neuroprotection could be advantageous in paediatric anaesthesia as there is growing concern, based on both laboratory studies and retrospective human clinical studies, that anaesthetics may trigger an injury in the developing brain, resulting in long-lasting neurodevelopmental consequences. Furthermore, xenon-mediated neuroprotection could help to prevent emergence delirium/agitation. Altogether, the beneficial haemodynamic profile combined with its putative organ-protective properties could render xenon an attractive option for anaesthesia of children undergoing cardiac catheterization.

**Methods/Design:**

In a phase-II, mono-centre, prospective, single-blind, randomised, controlled study, we will test the hypothesis that the administration of 50% xenon as an adjuvant to general anaesthesia with sevoflurane in children undergoing elective cardiac catheterization is safe and feasible. Secondary aims include the evaluation of haemodynamic parameters during and after the procedure, emergence characteristics, and the analysis of peri-operative neuro-cognitive function.

A total of 40 children ages 4 to 12 years will be recruited and randomised into two study groups, receiving either a combination of sevoflurane and xenon or sevoflurane alone.

**Discussion:**

Children undergoing diagnostic or interventional cardiac catheterization are a vulnerable patient population, one particularly at risk for intra-procedural haemodynamic instability. Xenon provides remarkable haemodynamic stability and potentially has cardio- and neuroprotective properties. Unfortunately, evidence is scarce on the use of xenon in the paediatric population. Our pilot study will therefore deliver important data required for prospective future clinical trials.

**Trial registration:**

EudraCT: 2014-002510-23 (5 September 2014)

## Background

Despite originally being described as chemically inert, noble gases including xenon, argon and helium have been repeatedly demonstrated to exhibit remarkable biological properties. In 1939, Behnke and Yarbrough observed narcotic effects of argon in humans at pressures from 4 to 10 atm [1]. In 1946, Lawrence reported anaesthetic properties of normobaric xenon in mice [2]. The first use of xenon for anaesthesia in humans was performed by Cullen and Gross in 1951 [2,3]. However, only the development of closed-circuit anaesthesia machines in the 1990s has made xenon available to a broader spectrum of patients. Xenon is described as having many of the characteristics of an ideal inhalational anaesthetic agent. It is non-flammable, non-explosive, non-toxic, devoid of teratogenic effects and does not contribute to the greenhouse effect. Due to the lowest blood/gas partition coefficient of all known anaesthetics, xenon provides rapid induction and emergence from anaesthesia. Moreover, xenon affects haemodynamics, myocardial performance and the neurohumoral system less than other anaesthetic agents [4].

Since the ‘re-introduction’ of xenon into clinical anaesthesia by Lachmann and colleagues in 1990 [5], xenon has been repeatedly demonstrated in experimental and clinical studies to produce only minimal haemodynamic side effects when compared to volatile or intravenous anaesthetics, even when used in heart failure conditions [6-9]. This inert haemodynamic profile may be owing to the fact that xenon anaesthesia is associated with significantly less sympathicolysis than known from other anaesthetics [10]. Meanwhile, these initial observations could be confirmed in two European multi-centre randomised controlled trials in which xenon was compared to isoflurane and found to slightly decrease heart rate and to preserve or moderately increase arterial pressures [11,12]. Moreover, recent evidence indicates that - in contrast to the majority of conventionally used general anaesthetics - xenon is virtually devoid of negative inotropic effects [12-15]. Furthermore, and adding to the culminating evidence that noble gases exert organ-protective effects in various conditions, xenon was found to induce both early and late pharmacological preconditioning in experimental models of myocardial ischemia [16].

Several clinical trials have been specifically performed in patients at increased cardiovascular risk or during cardiac surgery [17-20]. In this high-risk group, xenon anaesthesia was devoid of negative haemodynamic side effects, but instead showed a preservation of pre-anaesthetic haemodynamic parameters during the procedure.

The effects of xenon on right ventricular afterload are controversial. One study reported an increase in pulmonary arterial elastance in pigs that received xenon in addition to a baseline anaesthesia with pentobarbital and fentanyl [21]. In contrast, other investigators described xenon as not affecting pulmonary vascular resistance (PVR) in pigs [22]. Accordingly, Goto observed no changes in PVR in patients undergoing xenon anaesthesia [23]. Likewise, Bedi and co-workers found PVR did not change in critically ill patients who were sedated with xenon during their stay in the intensive care unit [24]. Our group also did not observe any effects of xenon on PVR in patients undergoing open-heart surgery [25].

From the present literature, there is hence no hint for a safety risk of xenon in patients with cardiac co-morbidity.

Apart from its beneficial effects on the cardiovascular system, xenon has been repeatedly demonstrated to offer neuroprotection in various animal models and models of neuronal injury, including neuronal ischemia, hypoxia, traumatic brain injury, neonatal asphyxia, cardiopulmonary bypass-associated neuronal injury and postoperative cognitive dysfunction (POCD) [26-33].

Xenon-mediated neuroprotection could be of particular interest in paediatric anaesthesia because epidemiologic studies have recently documented a correlation between anaesthetic exposure at a young age and subsequent learning difficulties and specific neurologic deficits [34-37]. Of note, xenon has been demonstrated to attenuate isoflurane-induced neuro-degeneration in rats [38,39]. In paediatric anaesthesia, exposure to sevoflurane is a well-known risk factor for the occurrence of emergence delirium after general anaesthesia in preschool children. Emergence delirium occurs in up to 80% of the anaesthetized children, has been associated with a prolonged stay in the postoperative care unit, can result in self-injury and has been linked to maladaptive behavioural changes during the late post-operative period. As the addition of xenon helps to reduce the sevoflurane concentrations and as the mechanism of anaesthesia of xenon is fundamentally different from that of sevoflurane, the incidence of emergence delirium could therefore be reduced [40].

The efficacy and safety of xenon for general anaesthesia has been repeatedly demonstrated in clinical trials, resulting in the medicolegal approval by the European Medicines Agency (EMA) in 2007.

Unfortunately, xenon has not been approved yet as an anaesthetic for children 0 to 18 years of age, most probably due to a paucity of clinical data that are required for a marketing approval. Up to now, there is no indication that xenon is associated with detrimental effects in children. In contrast, there is increasing evidence that xenon is also remarkably safe in the neonatal/paediatric population. Numerous studies have proven that xenon offers long-term functional and histopathologic neuroprotection in rodents after neonatal hypoxia/ischemia [26-28]. Also, in asphyxiated new-born pigs, xenon enhances hypothermic neuroprotection and offers stable haemodynamics [41,42]. Based on these animal data, two clinical trials are currently recruiting human neonates in the UK: one in which the effect of inhaled xenon (combined with therapeutic hypothermia) is evaluated in new-born infants with hypoxic-ischaemic encephalopathy (NCT01545271) and a second trial assessing whether following perinatal asphyxia, a combination therapy of inhaled xenon and hypothermia can preserve cerebral metabolism and structure (NCT00934700).

Up to now, the minimum alveolar concentration (MAC) of xenon required to produce anaesthesia in children is unknown. In adults, the MAC of xenon has been determined to be 63% [43]. It has to be assumed that - in accordance with other volatile or gaseous anaesthetics - the MAC of xenon in children is higher than in adults. Given a minimum requirement of 30% inhaled oxygen in ventilated patients, xenon mono-anaesthesia will be difficult to achieve in the paediatric population. Hence, xenon administration to children probably will have to be performed with sub-anaesthetic doses that will be applied in adjunction to the established anaesthetic sevoflurane. Such a strategy is associated with several advantages:

It has been repeatedly demonstrated that xenon interacts additively with other anaesthetics, which allows a significant dose reduction of the anaesthetics to which xenon is added and results in less haemodynamic compromise [44,45]. Further, in the majority of animal experiments, xenon has been demonstrated to exert its cardio- and neuroprotective effects already in sub-anaesthetic concentrations [0.25-0.5 MAC], suggesting that organ protection can be achieved independently from anaesthetic effects. In rats, it has been shown that the combination of xenon and sevoflurane preconditioning induces long-term neuroprotection in neonatal asphyxia [46]. Due to the scarcity of xenon (air contains only 87 ppb xenon), xenon anaesthesia is extremely costly. The combination of xenon with sevoflurane may be suited to reduce xenon consumption and associated costs without compromising xenon’s organ protective effects.

### Aims of the study

We hypothesise that combining sevoflurane anaesthesia with 50% xenon will be safe and feasible. Testing children undergoing cardiac catheterization (in which surgical trauma and inflammation is nearly absent) will also allow to study neuro-cognitive effects of anaesthetics independently from those induced by surgery.

## Methods/Design

### Design of the study

This phase II study is a mono-centre, prospective, single-blind, randomised and controlled trial.

The study will be performed according to the principles of the international declaration of Helsinki and to the principles of good clinical practice (GCP) and is approved by the ethics committee of the University Hospitals Leuven (S56902, 17 September 2014), by the Clinical Trials Centre of the University Hospital Leuven, and by the ‘Federaal Agentschap voor Geneesmiddelen en Gezondheidsproducten’. The study is registered in the European Clinical Trials Database of the European Medicines Agency (EudraCT Identifier: 2014-002510-23). Any eventual changes of the study protocol will be reported to the ethics committee.

Randomisation is being used to minimize selection bias. Patients will be randomised using a computer-generated permuted block randomisation sequence. Randomisation will be stratified by age (Stratum I: age 4 to 7 years; Stratum II: 8 to 12 years).

Concealment bias will be avoided by a masked randomisation procedure in which group assignments are ensured in sealed, opaque, sequentially numbered envelopes, which are opened only after the arrival of the patient in the catheterization room.

Due to the kind of intervention (administration of one or two anaesthetics via vapour and monitoring of xenon concentrations), the study has to be performed single-blind. The study will however be conducted by two investigator types: study enrolment and the post-anaesthesia follow-up will be performed by Investigator I who is (similarly to the patient) blinded to the study treatment, and Investigator II will only perform the general anaesthesia and will be, therefore, necessarily unblinded to the treatment conditions.

### Objectives

The primary objective of this study is to evaluate the safety and the feasibility of xenon anaesthesia in children. The safety will be assessed by the incidence of intraoperative adverse events (heart rate: >20% change from baseline; blood pressure: >20% change from baseline; requirements of vasopressors, inotropes and chronotropes). Moreover, we will compare average heart rate and blood pressure between both groups and also determine the average deviation of these parameters from baseline. The feasibility will be tested by intraoperative monitoring of depth of anaesthesia (physiologic signs plus bispectral index (BIS)) and by the intraoperative respiratory profile (as continuously measured by pulse oximetry and capnography).

Secondary endpoints of this study are as follow:

1. Invasive haemodynamic data, including intra-cardiac, systemic and pulmonary vascular pressures that are routinely measured during these procedures by the interventional cardiologist, after induction of anaesthesia and at the end of the procedure
2. Intraoperative fluid balance.
3. Intraoperative xenon/sevoflurane consumption.
4. Perioperative release of serum protein 100 β (S100 β) and interleukin (IL) 6.
5. Recovery times (measured from the stop of study treatment inhalation): time to open eyes, time to follow commands, time to extubation, time to (modified) Aldrete score ≥9 [47,48].
6. Incidence of emergence delirium as assessed with the ‘Paediatric Anaesthesia Emergence Delirium Scale’ [49] and the ‘Four-point Agitation Scale’ [47,50].
7. Incidence of post-operative nausea (PON) as assessed by a visual analogue score (VAS) in the post-anaesthesia care unit (PACU) and 12 to 24 h postoperatively. PON is defined as an unpleasant sensation associated with awareness of an urge to vomit [51]. The presence of nausea symptoms will be determined either by spontaneous reporting or by a direct inquiry made by the investigator ever 15 minutes throughout the PACU stay. The VAS nausea ranges from no nausea (score of 0) to extreme nausea (score of 100).
8. Incidence of post-operative vomiting (POV) in the post-anaesthesia care unit (PACU) and 12 to 24 h postoperatively. An emetic episode is defined as a single or continuing occurrence of vomiting or retching. Distinct episodes are defined by an interval of respite of more than 1 minute.
9. The duration of post-anaesthesia care unit and hospital stay.
10. Neuro-cognitive testing, 1 to 2 h and 12 to 24 h postoperatively (for details, see below).
11. Longitudinal follow-up of cognitive function after 1 year.
12. Incidence of (serious) adverse events ((S)AE) at any time point.

### Inclusion and exclusion criteria

The criteria for inclusion are children aged 4 to 12 years, who are scheduled for elective diagnostic or interventional cardiac catheterization under general anaesthesia and who are willing, along with their parents, to complete the requirements of this study, including the signature of the written informed consent.

The criteria for exclusion are cyanotic congenital heart defects possibly requiring a FiO2 of >40% during the procedure; high-risk and complex interventional procedures (as defined by the paediatric cardiologist); psychomotoric retardation (defined as the non-achievement of age-specific developmental milestones); or lack of informed consent from the subjects parents.

### Number of patients

A total of 40 children, 20 randomised in each of the two study groups, will be enrolled into this study.

This trial is planned as a pilot study to assess the safety and feasibility of xenon as an adjuvant to sevoflurane anaesthesia in children. A sample size calculation cannot be performed as required data are not available in literature. Given our experience with xenon trials in adults, a number of 40 patients should be sufficient to detect a statistically significant difference in safety and feasibility criteria [25].

### Investigational plan and treatments

Eligible patients will be randomly assigned to one of the two study groups. In group A, general anaesthesia will be maintained with xenon 50% in oxygen (FiO_2_ = 0.25 to 0.4) as an adjuvant to sevoflurane anaesthesia. In group B, general anaesthesia will be maintained with sevoflurane anaesthesia alone (FiO_2_ = 0.25 to 0.4). In both groups, sevoflurane end-tidal concentrations will be titrated according to the instantaneously registered EEG-monitoring in order to achieve a BIS-value between 40 and 60.

### Measurements and observations

See Figure 1.

**Figure 1:**
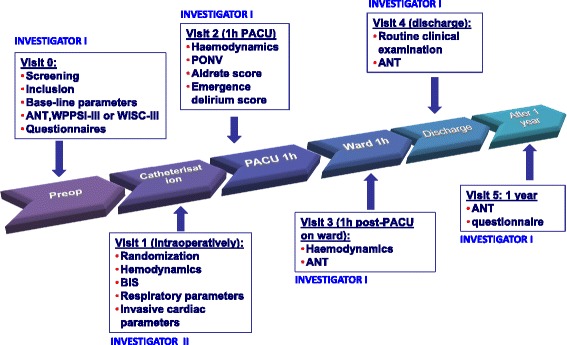
**Study visits.** ANT, Amsterdam Neuropsychological Tasks (ANT) battery; BIS, Bispectral index monitoring; PACU, Post-anaesthesia Care Unit; PONV, Postoperative nausea and vomiting; WPPSI, Wechsler Preschool and Primary Scale of Intelligence (>6y); WSCI, Wechsler Intelligence Scale for Children (<6y).

### Visit 0 (Investigator I)

#### Recruitment

Patients will be recruited by investigator I. Detailed information about the study background and the protocol will be given to the patients’ parents, and any possible questions brought forward by the parents will be answered. Parents of eligible children who agree to allow their child to participate in the study will sign a written informed consent before any specific study procedure will be initiated.

#### Pre-anaesthetic assessment

After obtaining the written informed consent, the investigator will evaluate and record baseline data (demographic data, medical and surgical history, and routine clinical examination) and perform baseline cognitive testing.

Cognitive function will be assessed by a selection of tests of the computerized Amsterdam Neuropsychological Tasks (ANT) battery designed for this study [52,53]. We will test simple motor reaction time, focused and divided attention, inhibitory control and cognitive flexibility and motor coordination. Parallel versions of the different ANT subtests will be used to minimize retest effects. The main advantage of the ANT is the combination of reaction time measurements and accuracy (error rate), which not only contributes to the sensitivity to detect problems in these neuro-cognitive domains but also can clarify whether the patient group predominantly suffers from deficits in processing speed, overall performance or a combination of both.

The outcomes of ANT will be adjusted to the general intellectual performance, registered by the assessment of the short version of the Wechsler Preschool and Primary Scale of Intelligence-III (WPPSI-III) for children younger than 6 years, and the Wechsler Intelligence Scale for Children (WISC-III) for patients between 6 and 16 years [54,55]. To complete the assessment of the baseline cognitive function, one of the parents will also be asked to complete two questionnaires at the first visit.

The first is a standardized questionnaire (120 items) for examining behavioural problems and competencies of children aged 4/6 to 18 (Child Behaviour Checklist (CBCL) 1½ -5 and 6–18 [56]). Internationally, this questionnaire is among the most used questionnaires and has been evaluated as adequate by the COTAN (‘Commissie Testaangelegenheden Nederland’) on all criteria [57].

This second questionnaire assesses executive functions of children by reports of parent (Behavioural Rating Inventory of Executive Functions (BRIEF)) [57]. It consists of eight subscales: inhibition, cognitive flexibility, regulation of emotions, taking initiative, working memory, planning and organising, orderliness, and monitoring of behaviour.

### Visit 1 (Investigator II)

#### General anaesthesia for cardiac catheterization

Patients must be in a fasting state for 6 h prior to anaesthesia. The children will not be premedicated with benzodiazepines, in order to avoid the well-known short-term impairment of cognitive function lasting for 48 h after midazolam premedication [58]. For non-pharmacological anxiolysis, parents will be encouraged to accompany their children until the induction of anaesthesia.

Patients will be continuously monitored and the following parameters will be recorded every 5 min from the pre-anaesthetic period to the end of the surgical procedure: arterial oxygen saturation (SaO_2_), non-invasive blood pressure (NIBP; systolic and diastolic blood pressure and mean arterial pressure), heart rate, FiO_2_, end-tidal O_2_ and CO_2_, temperature and BIS. Intra-cardiac, systemic and pulmonary vascular pressures will be measured twice during these procedures by the interventional cardiologist: after induction of anaesthesia and at the end of the procedure. At the same time points, 2 ml of blood will be obtained from the central venous sheet (*in situ* for the interventional procedure). After centrifugation this blood will be stored at −80°C for later determination of S100 β and IL-6.

#### Induction of anaesthesia

After a 2 min pre-oxygenation period (FiO2 = 1.0), general anaesthesia will be induced with a combination of: propofol 3 mg/kg IV as a bolus, fentanyl 2 μg/kg IV as a bolus and rocuronium 0.3 mg/kg IV as a bolus. Dexamethasone 0.15 mg/kg as a bolus IV will be given as a standard for PONV-prophylaxis.

If, in an exceptional case, an intravenous line is not available prior to induction, induction of anaesthesia will be performed by inhalation of sevoflurane.

#### Maintenance of anaesthesia

The administration of xenon 50% in oxygen (FiO2 = 0.25 to 0.4) as an adjuvant to sevoflurane anaesthesia (Group A) or sevoflurane anaesthesia alone (FiO2 = 0.25 to 0.4) (Group B) will be started once the patient has been intubated.

In both groups, sevoflurane end-tidal concentrations will be titrated according to the instantaneously registered EEG-monitoring in order to achieve a BIS-value between 40 and 60.

The investigational treatment will be administered until the end of the procedure. Then the application of xenon and sevoflurane will be stopped. Patients of both groups will be transferred when awake to the PACU.

#### Haemodynamic and ventilator management

Intraoperative haemodynamic management will be standardized according to our clinical routine. Systolic blood pressure and heart rate will be maintained within a 20% range of the baseline value. Deviations from this corridor will be treated by the administration of appropriate vasoactive and/or chronotropic medication. Ventilation will be adjusted to maintain end-tidal CO_2_ levels that reflect preoperative levels in these children.

#### Duration of treatment per patient

The duration of the treatment will be determined by the time from induction of anaesthesia until the end of the procedure. The average estimated time is 1 to 2 hours.

### Visit 2 (Investigator I): Post -anaesthesia study visit

This visit will be performed during the first 1 to 2 h after cardiac catheterization on the post-anaesthesia care unit (PACU). The following parameters will be assessed: routine clinical examination, including the assessment of heart rate and blood pressure; electrocardiogram; assessment of PON and POV; Aldrete score; and the incidence of emergence delirium.

If a patient with a VAS >20 complains of nausea, ondansetron 0.1 mg/kg will be administered immediately as a rescue medication.

Rescue medication for POV will be offered once the patient has more than one emetic episode. Rescue medication will also be offered on demand from the patient or his parents.

### Visit 3 (Investigator I): Post -PACU study visit on the ward

A neuro-cognitive examination (selection of tests of the ANT-battery designed for this study) will be performed 1 to 2 h after discharge from the PACU by investigator I.

### Visit 4 (Investigator I): Study end visit: The morning (12–24 h) following the procedure

On the morning following the cardiac catheterization, investigator I will perform a routine clinical examination, including the assessment of heart rate and blood pressure, an electrocardiogram and a final neuro-cognitive examination (tasks of the ANT battery as mentioned above). There will also be a last assessment of the incidence of PONV and (S)AE.

### Visit 5 (investigator I): Follow-up visit after 1 year

Neuro-cognitive examination (selection of tests of the ANT-battery designed for this study) will be repeated by investigator I one year after the cardiac catheterization and the parents will be asked to complete the BRIEF and CBCL questionnaire. This evaluation will take place in our institution at a routine follow-up with the paediatric cardiologist after one year, or if the child is not in follow-up in our institution anymore, an appointment will be made to visit the child at home.

### Data processing

Intraoperative data are recorded manually in 5 min intervals on specific case record forms (CRF). BIS-values will be electronically recorded every second. In addition, important pre- and postoperative data will be manually documented during the pre-specified visits on CRF’s.

### Statistical tests

An investigator or a study nurse will review completed CRFs for completeness and correctness before digitalization and statistical analysis. At this time point, missing data will be identified, if possible drawn from source data and filled into the CRFs. Missing data not being found in the source data will not be expected. In any case, data will be analysed according to the Intention-To-Treat principle.

All collected data will be analysed using descriptive statistics. After testing for normal distribution, primary and secondary outcome parameters will be compared between the two groups using appropriate comparative statistical methods.

A *P* <0.05 will be considered to indicate statistical significance in all employed tests.

Any deviations from the original statistical plan will be described and justified in the protocol and in the final report.

## Discussion

Children undergoing diagnostic or interventional cardiac catheterization are a vulnerable patient population that is at particular risk for intra-procedural haemodynamic instability. The ideal anaesthetic for these children should ensure excellent haemodynamic stability, without compromising oxygen delivery. Moreover it should maintain baseline haemodynamics to allow the assessment of intra-cardiac, systemic and pulmonary haemodynamics that are reflecting ‘true’ conditions, not affected by side effects of anaesthetics.

Xenon provides remarkable haemodynamic stability and potentially has cardio- and neuroprotective properties. Unfortunately, evidence is scarce on the use of xenon in the paediatric population. Our pilot study will provide data about the feasibility of xenon anaesthesia in children undergoing cardiac catheterization and will therefore deliver important data required to prospect larger clinical trials. We will focus on children age ≥4 years to enable reliable neuro-cognitive testing.

### Benefits for the participating patients

There is no guarantee that a combination of xenon and sevoflurane, instead of standard treatment with sevoflurane alone, will provide any medical advantage to the participant.

### Safety issues

The interventional treatment will be administered to patients with standard haemodynamic monitoring in the setting of a fully equipped cardiac catheterization room. This enables immediate detection and treatment of adverse events. Xenon inhalation will immediately be stopped in case the study patient shows a life-threatening deterioration. Also, after leaving the intervention room, all patients will be closely monitored by the study team for the occurrence of eventual (S)AE’s, first on the PACU and later on the normal ward. Moreover, the inclusion of each individual patient into the study is indicated in the electronic hospital information system and hence visible to all physicians and nurses involved in the care of this patient. This facilitates reporting of (S)AE’s to the principal investigator.

Moreover, study data will be regularly checked for safety by an independent clinical paediatric cardiologist who is not involved in the course of this clinical trial.

## Trial status

Patient recruitment will start in December 2014. The predicted study completion date is September 2016.

## Abbreviations

ANT: Amsterdam Neuropsychological Tasks
BIS: Bispectral index monitoring
BRIEF: Behavioural Rating Inventory of Executive Functions
CBCL: Child Behaviour Check List
COTAN: Commissie Test Aangelegenheden Nederland
CRF: case record form
EEG: electro-encephalogram
FiO2: fraction of inspired oxygen
IV: intravenous
MAC: minimum alveolar concentration
NIBP: non-invasive blood pressure
PACU: post-anaesthesia care unit
POCD: postoperative cognitive dysfunction
PON: post-operative nausea
PONV: post-operative nausea and vomiting
POV: post-operative vomiting
PVR: pulmonary vascular resistance
(S)AE: (serious) adverse event
SaO2: arterial oxygen saturation
WPPSI-III: Wechsler Preschool and Primary Scale of Intelligence for Children Younger Than 6 Years
WSCI-III: Wechsler Intelligence Scale for Children Between 6–16 Years

## References

[1] Yarbrough OD, Behnke AR (1939). The treatment of compressed air illness. J Ind Hyg Toxicol.

[2] Lawrence JH, Loomis WF (1946). Preliminary observations on the narcotic effect of xenon with a review of values for solubilities of gases in water and oils. J Physiol (Lond)..

[3] Cullen SC, Gross EG (1951). The anesthetic properties of xenon in animals and human beings, with additional observations on krypton. Science..

[4] Derwall M, Timper A, Kottmann K, Rossaint R, Fries M (2008). Neuroprotective effects of the inhalational anesthetics isoflurane and xenon after cardiac arrest in pigs. Crit Care Med..

[5] Lachmann B, Armbruster S, Schairer W, Landstra M, Trouwborst A, Van Daal GJ (1990). Safety and efficacy of xenon in routine use as an inhalational anaesthetic. Lancet..

[6] Boomsma F, Rupreht J, Man in ’t Veld AJ, Man in ’t Veld AJ, de Jong FH, Dzoljic M, Lachmann B (1990). Haemodynamic and neurohumoral effects of xenon anaesthesia. A comparison with nitrous oxide. Anaesthesia.

[7] Hettrick DA, Pagel PS, Kersten JR, Tessmer JP, Bosnjak ZJ, Georgieff M (1998). Cardiovascular effects of xenon in isoflurane-anesthetized dogs with dilated cardiomyopathy. Anesthesiology..

[8] Preckel B, Ebel D, Müllenheim J, Frässdorf J, Thämer V, Schlack W (2002). The direct myocardial effects of xenon in the dog heart in vivo. Anesth Analg.

[9] Preckel B, Schlack W, Heibel T, Rütten H (2002). Xenon produces minimal haemodynamic effects in rabbits with chronically compromised left ventricular function. Br J Anaesth..

[10] Neukirchen M, Hipp J, Schaefer MS, Brandenburger T, Bauer I, Winterhalter M (2012). Cardiovascular stability and unchanged muscle sympathetic activity during xenon anaesthesia: role of norepinephrine uptake inhibition. Br J Anaesth..

[11] Rossaint R, Reyle-Hahn M, Schulte Am Esch J, Scholz J, Scherpereel P, Vallet B (2003). Multicenter randomized comparison of the efficacy and safety of xenon and isoflurane in patients undergoing elective surgery. Anesthesiology.

[12] Wappler F, Rossaint R, Baumert J, Scholz J, Tonner PH, van Aken H (2007). Multicenter randomized comparison of xenon and isoflurane on left ventricular function in patients undergoing elective surgery. Anesthesiology..

[13] Schroth SC, Schotten U, Alkanoglu O, Reyle-Hahn MS, Hanrath P, Rossaint R (2002). Xenon does not impair the responsiveness of cardiac muscle bundles to positive inotropic and chronotropic stimulation. Anesthesiology..

[14] Hüneke R, Jüngling E, Skasa M, Rossaint R, Lückhoff A (2001). Effects of the anesthetic gases xenon, halothane, and isoflurane on calcium and potassium currents in human atrial cardiomyocytes. Anesthesiology..

[15] Baumert J-H, Hein M, Hecker KE, Satlow S, Neef P, Rossaint R (2008). Xenon or propofol anaesthesia for patients at cardiovascular risk in non-cardiac surgery. Br J Anaesth..

[16] Olsen EA, Brambrink AM (2013). Anesthesia for the young child undergoing ambulatory procedures: current concerns regarding harm to the developing brain. Curr Opin Anaesthesiol..

[17] Baumert J-H, Falter F, Eletr D, Hecker KE, Reyle-Hahn M, Rossaint R (2005). Xenon anaesthesia may preserve cardiovascular function in patients with heart failure. Acta Anaesthesiol Scand..

[18] Bein B, Turowski P, Renner J, Hanss R, Steinfath M, Scholz J (2005). Comparison of xenon-based anaesthesia compared with total intravenous anaesthesia in high risk surgical patients. Anaesthesia..

[19] Hanss R, Bein B, Turowski P, Cavus E, Bauer M, Andretzke M (2006). The influence of xenon on regulation of the autonomic nervous system in patients at high risk of perioperative cardiac complications. Br J Anaesth..

[20] Lockwood GG, Franks NP, Downie NA, Taylor KM, Maze M (2006). Feasibility and safety of delivering xenon to patients undergoing coronary artery bypass graft surgery while on cardiopulmonary bypass: phase I study. Anesthesiology..

[21] Hein M, Baumert J-H, Roehl AB, Pasch L, Schnoor J, Coburn M (2008). Xenon alters right ventricular function. Acta Anaesthesiol Scand..

[22] Marx T, Froeba G, Wagner D, Baeder S, Goertz A, Georgieff M (1997). Effects on haemodynamics and catecholamine release of xenon anaesthesia compared with total i.v. anaesthesia in the pig. Br J Anaesth.

[23] Goto T, Hanne P, Ishiguro Y, Ichinose F, Niimi Y, Morita S (2004). Cardiovascular effects of xenon and nitrous oxide in patients during fentanyl-midazolam anaesthesia. Anaesthesia..

[24] Bedi A, Murray JM, Dingley J, Stevenson MA, Fee JPH (2003). Use of xenon as a sedative for patients receiving critical care. Crit Care Med..

[25] Stoppe C, Fahlenkamp AV, Rex S, Veeck NC, Gozdowsky SC, Schälte G (2013). Feasibility and safety of xenon compared with sevoflurane anaesthesia in coronary surgical patients: a randomized controlled pilot study. Br J Anaesth..

[26] Zhuang L, Yang T, Zhao H, Fidalgo AR, Vizcaychipi MP, Sanders RD (2012). The protective profile of argon, helium, and xenon in a model of neonatal asphyxia in rats. Crit Care Med..

[27] Hobbs C, Thoresen M, Tucker A, Aquilina K, Chakkarapani E, Dingley J (2008). Xenon and hypothermia combine additively, offering long-term functional and histopathologic neuroprotection after neonatal hypoxia/ischemia. Stroke..

[28] Thoresen M, Hobbs CE, Wood T, Chakkarapani E, Dingley J (2009). Cooling combined with immediate or delayed xenon inhalation provides equivalent long-term neuroprotection after neonatal hypoxia-ischemia. J Cereb Blood Flow Metab..

[29] Deng J, Lei C, Chen Y, Fang Z, Yang Q, Zhang H (2014). Neuroprotective gases - Fantasy or reality for clinical use?. Prog Neurobiol..

[30] Höcker J, Stapelfeldt C, Leiendecker J, Meybohm P, Hanss R, Scholz J (2009). Postoperative neurocognitive dysfunction in elderly patients after xenon versus propofol anesthesia for major noncardiac surgery: a double-blinded randomized controlled pilot study. Anesthesiology..

[31] Vizcaychipi MP, Lloyd DG, Wan Y, Palazzo MG, Maze M, Ma D (2011). Xenon pretreatment may prevent early memory decline after isoflurane anesthesia and surgery in mice. PLoS One..

[32] Cremer J, Stoppe C, Fahlenkamp AV, Schälte G, Rex S, Rossaint R (2011). Early cognitive function, recovery and well-being after sevoflurane and xenon anaesthesia in the elderly: a double-blinded randomized controlled trial. Med Gas Res..

[33] Stuttmann R, Jakubetz J, Schultz K, Schäfer C, Langer S, Ullmann U (2010). Recovery index, attentiveness and state of memory after xenon or isoflurane anaesthesia: a randomized controlled trial. BMC Anesthesiol..

[34] Bong CL, Allen JC, Kim JTS (2013). The effects of exposure to general anesthesia in infancy on academic performance at age 12. Anesth Analg..

[35] DiMaggio C, Sun LS, Li G (2011). Early childhood exposure to anesthesia and risk of developmental and behavioral disorders in a sibling birth cohort. Anesth Analg..

[36] Stratmann G (2011). Review article: Neurotoxicity of anesthetic drugs in the developing brain. Anesth Analg..

[37] Sun L (2010). Early childhood general anaesthesia exposure and neurocognitive development. Br J Anaesth..

[38] Ma D, Williamson P, Januszewski A, Nogaro M-C, Hossain M, Ong LP (2007). Xenon mitigates isoflurane-induced neuronal apoptosis in the developing rodent brain. Anesthesiology..

[39] Shu Y, Patel SM, Pac-Soo C, Fidalgo AR, Wan Y, Maze M (2010). Xenon pretreatment attenuates anesthetic-induced apoptosis in the developing brain in comparison with nitrous oxide and hypoxia. Anesthesiology..

[40] Dahmani S, Delivet H, Hilly J (2014). Emergence delirium in children: an update. Curr Opin Anaesthesiol..

[41] Chakkarapani E, Dingley J, Liu X, Hoque N, Aquilina K, Porter H (2010). Xenon enhances hypothermic neuroprotection in asphyxiated newborn pigs. Ann Neurol..

[42] Chakkarapani E, Thoresen M, Liu X, Walloe L, Dingley J (2012). Xenon offers stable haemodynamics independent of induced hypothermia after hypoxia-ischaemia in newborn pigs. Intensive Care Med..

[43] Nakata Y, Goto T, Ishiguro Y, Terui K, Kawakami H, Santo M (2001). Minimum alveolar concentration (MAC) of xenon with sevoflurane in humans. Anesthesiology..

[44] Goto T, Nakata Y, Ishiguro Y, Niimi Y, Suwa K, Morita S (2000). Minimum alveolar concentration-awake of Xenon alone and in combination with isoflurane or sevoflurane. Anesthesiology..

[45] Barakat AR, Schreiber MN, Flaschar J, Georgieff M, Schraag S (2008). The effective concentration 50 (EC50) for propofol with 70% xenon versus 70% nitrous oxide. Anesth Analg.

[46] Luo Y, Ma D, Ieong E, Sanders RD, Yu B, Hossain M (2008). Xenon and sevoflurane protect against brain injury in a neonatal asphyxia model. Anesthesiology..

[47] Cho EJ, Yoon SZ, Cho JE, Lee HW (2014). Comparison of the Effects of 0.03 and 0.05 mg/kg Midazolam with Placebo on Prevention of Emergence Agitation in Children Having Strabismus Surgery. Anesthesiology..

[48] Aldrete JA (1995). The post-anesthesia recovery score revisited. J Clin Anesth..

[49] Sikich N, Lerman J (2004). Development and psychometric evaluation of the pediatric anesthesia emergence delirium scale. Anesthesiology..

[50] Aono J, Ueda W, Mamiya K, Takimoto E, Manabe M (1997). Greater incidence of delirium during recovery from sevoflurane anesthesia in preschool boys. Anesthesiology..

[51] Gan TJ, Diemunsch P, Habib AS, Kovac A, Kranke P, Meyer TA (2014). Consensus guidelines for the management of postoperative nausea and vomiting. Anesth Analg..

[52] de Sonneville L (2011). Amsterdam Neuropsychological Tasks: Manual (Handleiding).

[53] Mesotten D, Gielen M, Sterken C, Claessens K, Hermans G, Vlasselaers D (2012). Neurocognitive development of children 4 years after critical illness and treatment with tight glucose control: a randomized controlled trial. JAMA..

[54] Vander Steene G, Bos A (1997). WPSSI-R: Vlaams-Nederlandse Aanpassing.

[55] Wechsler D (2004). WAIS III, Nederlandstalige Bewerking: Afname en Scoringshandleiding.

[56] Verhulst FC, van der Ende J, Koot JM (1996). Handleiding Voor De CBCL/4-18.

[57] Smidts DP, Huizinga M (2009). BRIEF Executieve Functies Gedragsvragenlijst: Handleiding.

[58] Millar K, Asbury AJ, Bowman AW, Hosey MT, Martin K, Musiello T (2007). A randomised placebo-controlled trial of the effects of midazolam premedication on children’s postoperative cognition. Anaesthesia..

